# Association of *Chlamydia trachomatis* infection with pregnancy outcomes among Japanese pregnant women: The Japan environment and children’s study

**DOI:** 10.1371/journal.pone.0275573

**Published:** 2022-11-29

**Authors:** Shun Yasuda, Hyo Kyozuka, Yuta Endo, Aya Kanno, Tsuyoshi Murata, Toma Fukusda, Akiko Yamaguchi, Akiko Sato, Yuka Ogata, Masahito Kuse, Mitsuaki Hosoya, Seiji Yasumura, Koichi Hashimoto, Hidekazu Nishigori, Keiya Fujimori, Japan Environment, Children’s Study JECS Group

**Affiliations:** 1 Fukushima Regional Center for the Japan Environmental and Children’s Study, Fukushima, Japan; 2 Department of Obstetrics and Gynecology, Fukushima Medical University School of Medicine, Fukushima, Japan; 3 Department of Pediatrics, Fukushima Medical University School of Medicine, Fukushima, Japan; 4 Department of Public Health, Fukushima Medical University School of Medicine, Fukushima, Japan; 5 Fukushima Medical Center for Children and Women, Fukushima Medical University, Fukushima, Japan; West China Second University Hospital of Sichuan University, CHINA

## Abstract

This study aimed to investigate the impact of *Chlamydia trachomatis* (CT) infection on pregnancy outcome in pregnant Japanese women. We utilized the data from a nationwide birth cohort study, the Japan Environment and Children’s Study (JECS), for this study. We enrolled 26,385 individuals who could refer to data on pregnancy outcomes and confounding factors, with data on CT. Binominal logistic regression models were used to determine whether pregnant women with CT positivity were at more risk of experiencing adverse pregnancy outcomes, preterm birth (PTB), preterm prelabor rupture of membrane (pPROM), low birth weight (LBW) infants, small for gestational age (SGA) births, or hypertensive disorders of pregnancy (HDP). After adjusting for maternal age, parity, marital status, smoking status, and education status, there were no significantly increased risks of PTB, pPROM, LBW infants, SGA, and HDP in the odds ratios. No significant increase in the risk of adverse pregnancy outcomes was observed in any of the subgroup analyses, which were limited to the pregnancy women in Fukushima prefecture, where CT screening could be confirmed at 28−30 weeks of gestation. We believe that the results of this study will make a significant contribution to the future of medical care for pregnant women in Japan. Our findings are important for medical practitioners to contribute to the future medical treatment of Japanese pregnant women, and also to contribute to pre-conception care for Japanese society as a whole, including pregnant women.

## Introduction

Genital *Chlamydia trachomatis* (CT) infection is the most common sexually transmitted disease in Japan [1]. The purpose of screening pregnant women for CT in Japan is to prevent mother-to-child transmission of CT. Neonates born vaginally in women with cervicitis due to CT acquire CT at a rate of approximately 50−70%; approximately 20−50% of neonates who acquire CT develop chlamydial conjunctivitis and 30% develop chlamydia pneumonia [2–10]. Therefore, to prevent the development of neonatal chlamydial infection, pregnant women should be screened for chlamydial cervicitis, even if few clinical signs are present [11, 12].

In 2010, the Japanese Ministry of Health, Labour and Welfare announced subsidized chlamydia testing during maternal health screening. Since 2011, pregnant women in Japan have been subjected to universal screening for CT.

A set of Japanese guidelines is available for obstetricians regarding management of CT in clinical practice [13]; the guidelines state that genital CT infection can induce chorioamnionitis and may be considered a risk factor for adverse pregnancy outcomes, including preterm birth (PTB), preterm prelabor rupture of membrane (pPROM), and small for gestational age (SGA) births; however, no studies on large maternal populations have been performed investigating the association of CT with pregnancy outcomes since the universal screening for CT had been implemented in Japan. This is the first known report using a nationwide birth cohort study investing CT in pregnancy and pregnancy outcomes in Japan.

## Materials and methods

### Study design

In the present study, we used data from the Japan Environment and Children’s Study (JECS), which is a nationwide and government-funded birth cohort study that was started in January 2011 to investigate the effects of environmental factors on children’s health [14, 15]. The eligibility criteria for JECS participants (expecting mothers) were as follows: (1) residing in the study areas at the time of recruitment and expected to reside continually in Japan for the foreseeable future, (2) expected delivery date between August 1, 2011 and mid-2014, and (3) capable of participating in the study without difficulty (i.e., ability to comprehend the Japanese language and complete the self-administered questionnaire).

The target recruitment rate was more than 50% of all eligible mothers. Either or both of the following two recruitment protocols were applied: (1) recruitment at the time of the first prenatal examination by co-operating health care providers and/or (2) recruitment at local government offices issuing pregnancy journals, namely the *Mother-Child Health Handbook*, which is provided to all expecting mothers in Japan before receiving municipal services for pregnancy, delivery, and childcare. Written informed consent was obtained from all participants.

The JECS protocol was reviewed and approved by the Ministry of the Environment’s Institutional Review Board on Epidemiological Studies and by the Ethics Committees of all participating institutions (The National Center for Child Health and Development, National Institute for Environmental Studies, Hokkaido University, Sapporo University, Asahikawa Medical College, Japanese Red Cross Hokkaido College of Nursing, Tohoku University, Fukushima Medical University, Chiba University, Yokohama City University, University of Yamanashi, Shinshu University, University of Toyama, Nagoya City University, Kyoto University, Doshisha University, Osaka University, Osaka Medical Center and Research Institution for Maternal and Child Health, Hyogo College of Medicine, Tottori University, Kochi University, Kyushu University, University of Occupational and Environmental Health, Kumamoto University, University of Miyazaki, and University of Ryukyu). The JECS was conducted in accordance with the Helsinki Declaration and other nationally valid regulations and guidelines.

### Data collection

Data for the current analysis used the data set released in June 2016 (data set: jecs-ag-20160424). In this data set, we used three types of data: (1) MT1, obtained from a self-reported questionnaire collected during their first trimester (the first questionnaire) that included questions regarding maternal medical background; (2) MT2, obtained from a self-reported questionnaire collected during their second/third trimester (second questionnaire) that included information on the lifestyle and socioeconomic status; (3) Dr-0m, which included obstetric outcomes, such as gestational age, birth weight, and cervical chlamydia test results, collected from medical record transcripts provided by each subject’s co-operating health care provider. We excluded multiple pregnancies (twins and triplets) and cases with inadequate data. We integrated each data set used in JECS MT1, MT2, and Dr-0m, relying on the number assigned to the participant.

### Pregnancy outcomes, and confounding factors

Pregnancy outcome data included gestational age at birth, pPROM, and birth weight. PTB was classified into two categories: before 37 weeks and before 32 weeks. pPROM was defined as membrane rupture before 37 weeks of gestation. Low birth weight (LBW) was defined as birthweight below 2,500 g. SGA was defined as a birth weight below −1.5 standard deviation corrected for gestational age and sex according to the New Japanese neonatal anthropometric charts for gestational age at birth [16]. We defined HDP in this study as a systolic blood pressure of ≧140 mmHg and/or a diastolic blood pressure of ≥90 mmHg that developed after conception based on the methods we have already published in HDP using this data in the past [17]. The five primary outcomes therefore were SGA, pPROM, PTB, LBW and HDP.

The values analyzed as potential confounding factors during pregnancy were maternal age at delivery, maternal body mass index (BMI) before pregnancy, parity, maternal smoking status, marital status, and maternal educational status. Maternal age at delivery and BMI were each categorized into three groups: age ≤19, 20–34, and ≥35 years [18–27] and BMI <18.5, 18.5−25.0, and >25.0 kg/m^2^ [18–30]. Maternal participants in questionnaire MT2 were requested to provide information about their smoking history and current marital status. The questions about smoking in the questionnaire were: “kept smoking during pregnancy,” “never smoked,” “quit smoking before pregnancy,” and “quit smoking during early pregnancy.” The maternal participants who chose “kept smoking during pregnancy” were classified into the smoking category and the others into the non-smoking. The options on marital status in the questionnaire were: “currently married,” “not married ever,” “divorced and not married now,” “husband died and not married now.” The maternal participants who chose “currently married” were classified into the “married” category and the others into the “unmarried” category. The highest level of education was categorized into four groups: junior high school: <10 years; high school, technical junior college: 10–12 years; technical/vocational college, associate degree, Bachelor’s degree: 13–16 years; and graduate degree (Master’s/Doctor’s): ≥17 years [18, 20–23, 27–30].

### Diagnosis of CT infection

In Japan, CT screening at 30 weeks of gestation is financially subsidized. We obtained and analyzed the results of this nationwide screening, and found that some municipalities in Japan perform CT screening at the first trimester or at other periods during the pregnancy (reference in English is not available). In this study, we selected the results of the CT screening in the Fukushima prefecture from the total participants since it is widely known that in Fukushima prefecture the screenings are only performed at 28−30 weeks of gestation.

We obtained the results of these screening tests. CT infection is diagnosed by detecting CT in cervical secretions or brushed specimens. The Japanese guidelines for obstetricians [13] state that detecting antibodies for CT is not sufficient to screen for CT.

Therefore, each co-operating health care provider uses highly sensitive nucleic acid amplification methods, such as transcription mediated amplification, polymerase chain reaction (PCR), or strand displacement amplification, and the less sensitive enzyme immunoassay and nucleic acid detection tests are rarely used.

### Statistical analysis

Since the continuous variables between CT positive and CT negative participant characteristics used in this study were not normally distributed in the Kolmogorov–Smirnov test of normality, the participant characteristics were analyzed using the Mann–Whitney U test for comparison. The chi-square test or Fisher’s exact test was used to compare categorical variables.

To evaluate the impact of CT infection on pregnancy outcomes, the crude odds ratio (cOR), adjusted odds ratio (aOR) from a single multivariate model, and 95% confidence interval (95% CI) were also analyzed using binominal logistic regression analysis.

SPSS version 26 (IBM Corp., Armonk, NY, USA) was used for the statistical analysis. A P-value < 0.05 was considered statistically significant.

## Results

**Fig 1:**
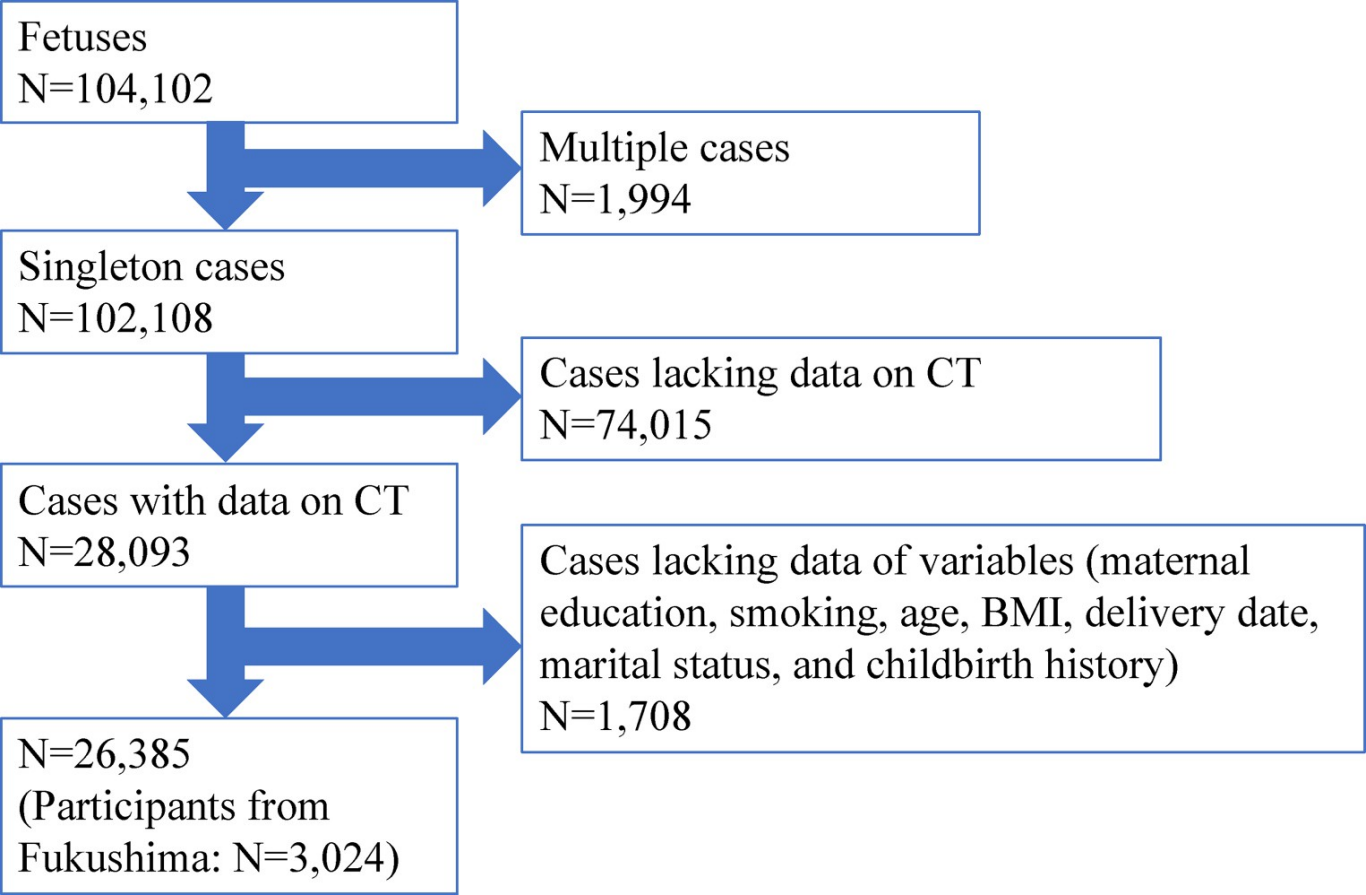
Flowchart of the recruitment of study participants. On application of the eligibility criteria, 26,385 mothers were considered to be eligible from the data obtained on 104,102 fetuses from the JECS. Of these, 3,024 cases from the Fukushima prefecture were enrolled in this study. Abbreviations: CT: *Chlamydia trachomatis*; BMI: body mass index.

Between August 1, 2011 and mid-2014, data on 104,102 fetuses were obtained from the JECS. After applying our inclusion criteria, 26,385 maternal participants were considered eligible for the study. Of these, the number of participants from the Fukushima prefecture was 3,024 (Fig 1).

### Maternal medical and socioeconomic backgrounds and pregnancy outcomes

The total number of participants was 26,385. Table 1 shows the maternal medical and socioeconomic background data and the pregnancy outcomes of all participants. The number of participants positive on CT screening was 1,196 (4.5%).

**Table 1:** Maternal medical and socioeconomic backgrounds and pregnancy outcomes (total participants).

Variable	CT negative (n = 25,189)	CT positive (n = 1,196)	p-value
Maternal age, years, median (IQR)	31.0 (24.0−38.0)	27.0 (19.0−35.0)	<0.001‡
Maternal age, years, n (%)			<0.001*
≦19	206 (70.8)	85 (29.2)	
20−34	17,999 (94.9)	959 (5.1)	
≧35	6,984 (97.9)	152 (2.1)	
BMI before pregnancy (kg/m^2^), median (IQR)	20.6 (17.2–24.0)	20.5 (16.8–24.2)	0.199‡
BMI before pregnancy (kg/m^2^), n (%)			0.007*
<18.5	4,099 (94.6)	232 (5.4)	
18.5–25.0	18,417 (95.7)	827 (4.3)	
>25.0	2,673 (95.1)	137 (4.9)	
Nulliparous, n (%)	9,877 (39.2)	716 (59.9)	<0.001*
Smoking during pregnancy, n (%)	1,143 (4.9)	103 (9.7)	<0.001*
Unmarried, n (%)	1,086 (4.3)	203 (17.0)	<0.001*
Maternal education, n (%)			<0.001*
<10 years	1221 (87.8)	170 (12.2)	
10–12 years	7825 (94.0)	501 (6.0)	
13–16 years	15781 (96.8)	518 (3.2)	
>17 years	362 (98.1)	7 (1.9)	
Gestational weeks at birth, median (IQR)	39.0 (37.0–41.0)	39.0 (37.0–41.0)	<0.001‡
PTB <37 weeks, n (%)	1,152 (4.6)	49 (4.1)	0.440*
PTB <32 weeks, n (%)	117 (0.5)	5 (0.4)	1.000†
pPROM, n (%)	330 (1.3)	15 (1.3)	0.985†
LBW<2,500 g, n (%)	1921 (7.6)	98 (8.2)	0.475*
SGA, n (%)	1,246 (4.6)	65 (5.4)	0.448*
HDP, n (%)	791 (3.1)	47 (3.9)	0.128*

*Chi-square test

†Fisher’s exact test

‡ Mann−Whitney U test

BMI: body mass index; PTB: preterm birth; pPROM: preterm prelabor rupture of membrane; LBW: low birth weight infant; SGA: small-for gestational age; HDP: hypertension disorder of pregnancy; IQR: interquartile range

The mean maternal age was lower in the CT positive than in the CT negative group; the difference was statistically significant. The categorized maternal age also showed a statistically significant difference. The rate of chlamydia infection was 27.3% in those aged ≤19 years, 5.1% in those aged 20−34 years, and 2.1% in those aged ≥35 years. Mean BMI did not show a statistically significant difference between the two groups, whereas there was a significant difference in categorized BMI. Categorized maternal educational states also showed a significant difference. The proportion of nulliparous women, women smoking during pregnancy, and unmarried women was significantly higher in those who were CT positive than in those who were CT negative.

The difference in the median gestational age at delivery was statistically significant, but the ratio of PTB <37 weeks of gestation, <32 weeks of gestation showed no statistical significance. The ratio of LBW<2,500 g, SGA, and HDP also did not show any statistical significance between the two groups.

Of the total participants, 3,024 participants were from Fukushima prefecture. Of these, 4.6% were CT positive (135 participants) (Table 2).

**Table 2:** Maternal medical and socioeconomic backgrounds and pregnancy outcomes (Fukushima participants).

Variable	Fukushima CT negative (n = 2,889)	Fukushima CT positive (n = 135)	p-value
Maternal age, years, median (IQR)	30.0 (23.0–37.0)	27.0 (18.0–37.0)	<0.001‡
Maternal age, years, n (%)			<0.001*
≦19	16 (66.7)	8 (33.3)	
20−34	2,203 (95.1)	113 (4.9)	
≧35	670 (98.0)	14 (2.0)	
BMI before pregnancy (kg/m^2^), median (IQR)	20.8 (17.3–24.3)	20.7 (16.8–24.6)	0.130‡
BMI before pregnancy (kg/m^2^), n (%)			<0.001*
<18.5	413 (92.0)	36 (8.0)	
18.5−25.0	2,123 (96.4)	80 (3.6)	
>25.0	353 (94.9)	19 (5.1)	
Nulliparous, n (%)	1,106 (38.3)	87 (64.4)	<0.001†
Smoking during pregnancy, n (%)	102 (3.5)	10 (7.4)	0.031†
Unmarried, n (%)	108 (3.7)	21 (15.6)	<0.001†
Maternal education, n (%)			0.030*
<10 years	115 (92.0)	10 (8.0)	
10–12 years	1147 (94.5)	67 (5.5)	
13–16 years	1610 (96.6)	57 (3.4)	
>17 years	17 (94.4)	1 (5.6)	
Gestational weeks at birth, median (IQR)	39.0 (37.0–41.0)	39.0 (37.0–41.0)	0.074‡
PTB <37 weeks, n (%)	134 (4.6)	3 (2.2)	0.285†
PTB <32 weeks, n (%)	7 (0.2)	0 (0)	1.000†
pPROM, n (%)	34 (1.2)	0 (0)	0.402†
LBW<2,500 g, n (%)	216 (7.5)	11 (8.1)	0.738†
SGA, n (%)	154 (5.3)	10 (7.4)	0.326†
HDP, n (%)	76 (2.6)	6 (4.4)	0.180†

*Chi-square test

†Fisher’s exact test

‡ Mann−Whitney U test

BMI: body mass index; PTB: preterm birth; pPROM: preterm prelabor rupture of membrane; LBW: low birth weight infant; SGA: small-for gestational age; HDP: hypertension disorder of pregnancy; IQR: interquartile range

Maternal characteristics, such as maternal age, BMI, parity, smoking status, marital status, and educational status, were compared between CT negative and CT positive participants from Fukushima prefecture. The results were similar to those obtained from comparing the total participants. The results of comparing pregnancy outcomes between the two groups were also similar to the results obtained from comparing the total participants. Among participants from Fukushima, CT positive had no pPROMs or PTB<32 weeks of gestation.

### Effect of CT infection on pregnancy outcomes

Table 3 shows the association of CT infection with each pregnancy outcome. cOR of PTB<37 weeks of gestation, PTB<32 weeks of gestation, pPROM, LBW<2,500 g, and SGA showed that CT positive had no effect on the pregnancy outcomes. The aORs (95% CI) of PTB<37 weeks of gestation, PTB<32 w gestation, pPROM, LBW<2,500 g, SGA, and HDP were 0.88 (0.65–1.18), 0.74 (0.30–1.85), 0.90 (0.53–1.53), 1.01 (0.82–1.26), 1.08 (0.83–1.40), and 1.18 (0.87–1.60), which also showed that CT positive did not affect the outcomes.

**Table 3:** Effect of CT infection on pregnancy outcomes (total participants).

	PTB<37 weeks	PTB<32 weeks	pPROM	LBW<2,500 g	SGA	HDP
cOR (95% CI)	0.89 (0.67–1.19)	0.90 (0.37–2.21)	0.96 (0.57–1.61)	1.08 (0.87–1.36)	1.10 (0.85–1.43)	1.26 (0.94–1.70)
aOR (95% CI)	0.88 (0.65–1.18)	0.74 (0.30–1.85)	0.90 (0.53–1.53)	1.01 (0.82–1.26)	1.08 (0.83–1.40)	1.18 (0.87–1.60)

CT positive (reference: CT negative)

CT: *Chlamydia trachomatis*, PTB: preterm birth, pPROM: preterm pre-labor rupture of membrane, LBW: low birth weight, SGA: small for gestational age, HDP: hypertension disorder of pregnancy; cOR: Crude odds ratio, aOR: Adjusted odds ratio. aOR is adjusted for maternal age, parity, marital status, smoking status, and education. 95% CI: 95% confidence interval

Similarly, no significant difference was seen in cOR or aOR from the results of the participants from Fukushima (Table 4).

**Table 4:** Effect of CT infection on pregnancy outcomes (Fukushima participants).

	PTB<37 weeks	PTB<32 weeks	pPROM	LBW<2,500 g	SGA	HDP
cOR (95% CI)	0.47 (0.15–1.49)	N/A	N/A	1.10 (0.58–2.07)	1.42 (0.73–2.76)	1.72 (0.74–4.03)
aOR (95% CI)	0.47 (0.15–1.51)	N/A	N/A	1.04 (0.55–1.98)	1.31 (0.66–2.58)	1.76 (0.74–7.20)

CT positive (reference: CT negative)

CT: *Chlamydia trachomatis*, PTB: preterm birth, pPROM: preterm prelabor rupture of membrane, LBW: low birth weight, SGA: small for gestational age, HDP: hypertension disorder of pregnancy; N/A.: Data not available, cOR: crude odds ratio, aOR: adjusted odds ratio, 95% CI: 95% confidence interval

aOR is adjusted for maternal age, parity, marital status, smoking status, and education.

## Discussion

To the best of our knowledge, this is the first study on CT among Japanese pregnant women using JECS, which provides a large sample size of well-documented participants.

While the Japanese guidelines for obstetricians state that CT infection can affect pregnancy outcomes, especially in preterm labor, this has not been investigated on a large scale in Japan, and we believe that this important question has been clarified in this study. The results of this study showed no association between CT infection and adverse pregnancy outcomes, namely preterm delivery at <37 or <32 weeks, LBW<2,500 g, SGA, or HDP.

Recently, the association between CT infection and PTB was reported in a large population-based study including more than 20,000 pregnant women from Australia; the authors found no association between CT infection during pregnancy and PTB or SGA [31].

A recent single-center retrospective study from Japan showed that CT infection had no adverse effects on pregnancy outcome, which is consistent with our results [32].

On the other hand, as stated in the guidelines issued for Japanese obstetricians [13], there are many studies reporting that CT infection may adversely affect pregnancy outcomes, including PTB. In fact, there are reports that CT infection was associated with an increase in PTB, pPROM, LBW births, or SGA [33–35]; however, different studies have reported conflicting results. One possible reason for these ambiguous results is the lack of agreement on the definition of PTB. While it is important to distinguish between spontaneous and artificial PTBs, the fact that these two outcomes are sometimes not distinguished has been identified as problematic. Although our study is large in scope, it is difficult to distinguish strictly between spontaneous and artificial PTBs. The major cause of artificial PTB is HDP, and HDP is highly related to SGA. We found that CT infection did not increase HDP, nor was it associated with an increased frequency of SGA. Although there are several other reasons for artificial PTB, we consider that CT infection would not have increased artificial PTB, on considering its associations with HDP, SGA, or LBW.

In Japan, when CT is detected in the cervix, according to obstetric guidelines [13], a single dose of azithromycin (1.0 g) or clarithromycin (200 mg × 2/day for 7 days) is administered to prevent neonatal CT infection and complete treatment before delivery. This may have contributed to preventing an increase in PTBs or pPROMs due to CT infection in Japan. However, a double-blind, randomized, controlled trial on pregnant women with chlamydial infection found no difference in the rate of PTBs or pPROMs between the antibiotic-treated and placebo groups [36]. Antimicrobial treatment for CT during pregnancy has been reported to be safe [18]; however, it should be noted that treatment of CT infection during pregnancy may not change the risk of PTB or pPROM.

It is difficult to identify when CT screening was performed and what treatment was administered to CT positive patients based only on the questionnaire. This is because the only CT data available from the Dr0M data used in this study are the positive or negative results of the patient’s chlamydia screening test. Therefore, we conducted a subgroup analysis of participants from Fukushima prefecture to which our author belongs in the present study. In Fukushima prefecture, the overall policy is to perform CT screening at 28–30 weeks of gestation. CT is an infection that can cause a “ping-pong infection” between partners; even if treated, there is a risk of re-infection during the same pregnancy. The timing of 28–30 weeks of gestation is the latest period in pregnancy when a pregnant woman can be found positive, treated, and delivered shortly after a negative test has been confirmed. Interestingly, we found no evidence of an effect of CT positive on PTB or other adverse pregnancy outcomes in the participants, who would have been screened at various times of the year pregnancy, or in the Fukushima prefecture study alone. It is very interesting to consider that the results support the possibility that CT does not affect PTB, even if the timing of screening or treatment is different during pregnancy. The CT positive patients recruited for this study are likely to have been treated; however, it has never been proven that treating CT positive also prevents PTB. Although this study cannot say whether the presence or absence of treatment prevented the association with PTB, it does seem to indicate that appropriate intervention by screening for chlamydia at any time during pregnancy in Japan does not affect pregnancy outcome; however, it might also be that the treatment prevents women from having adverse pregnancy outcomes.

To the best of our knowledge, this is the first study to analyze the association between CT infection and pregnancy outcomes from a large nationwide birth cohort study of JECS with 26,385 women, which enrolled the largest number of participants in Japan. However, several limitations need to be considered. The method of diagnostic testing used for CT infection is unclear, as this information was not requested in the questionnaire. A survey conducted in 2013–2014 found that among 1,644 Japanese obstetric facilities, CT nucleic acid detection using PCR was performed in 1,221 (74.3%), CT nucleic acid detection using methods other than PCR was performed in 408 (24.8%), and CT antibody detection was performed in 15 (0.9%) [1]. However, no data for the time period of that study exists. In addition, while information on 104,102 (102,108 single fetuses) were acquired in total, there was missing information on chlamydia testing. This may be because the timing of our study coincided with the start of universal screening for chlamydia in Japan around 2011. Information on treatment of chlamydia during pregnancy, chlamydia-related complications in neonates, and co-occurrence of other sexually transmitted diseases was difficult to obtain from the JECS questionnaire, and a larger scale specific survey focusing on sexually transmitted infections, including CT, is needed to resolve these limitations. The data used in this study is largely based on self-reported data. Self-reporting is one of the information biases and has a tendency to underreport. Although self-reporting is an easy way to collect information such as smoking information, the possibility that data far from the truth may be obtained is a limitation.

Based on our study results, it is likely that chlamydia infection is closely related to the socioeconomic background. A detailed analysis of this correlation is also important and should be conducted separately from the present study, which focused on the relationship between CT infection and pregnancy outcomes.

## Conclusion

We found no association between CT infection and adverse pregnancy outcomes. We believe that the results of this study will make a significant contribution to the future of medical care for pregnant women in Japan.

Our findings are important for medical practitioners to contribute to the future medical treatment of Japanese pregnant women, and also to contribute to pre-conception care for Japanese society as a whole, including pregnant women.
